# Supplementary material for: Controlled Synthesis of Au25 Superatom Using a Dendrimer Template
Source: Molecules. 2022 May 25;27(11):3398. doi: 10.3390/molecules27113398 (PMC9182415; doi:10.3390/molecules27113398)
Supplement: Supplementary file 1 [file molecules-27-03398-s001.zip › molecules-1714718-supplementary.pdf]

molecules

Supporting Information

Controlled synthesis of Au_{25} superatom using a dendrimer template

Hisanori Muramatsu [1], Tetsuya Kambe [1,2], Takamasa Tsukamoto [1,2,3], Takane Imaoka [1,2] and Kimihisa Yamamoto [1,2,*]

[1] Laboratory of Chemistry and Life Science (CLS), Tokyo Institute of Technology, Yokohama 226-8503, Japan; muramatsu.h.ae@m.titech.ac.jp (H.M.); kambe.t.aa@m.titech.ac.jp (T.K.); tsukamoto.t.ad@m.titech.ac.jp (T.T.); timaoka@res.titech.ac.jp (T.I.); yamamoto.k.at@m.titech.ac.jp (K.Y.)
[2] JST-ERATO, Yamamoto Atom Hybrid Project, Tokyo Institute of Technology, Yokohama 226-8503, Japan
[3] JST-PRESTO, Kawaguchi, Saitama, 332-0012, Japan
* Correspondence: yamamoto.k.at@m.titech.ac.jp (K.Y.); Tel.: +81-45-924-5260 (K.Y.)

Supporting Figure List

Figure S1. Schematic diagram of the structure and coordination of the dendrimer (TPM G4) used in this study.

Figure S2. (a) Coordination image of $AuCl_3$ to TPMG4, (b) UV-Vis absorption spectra obtained by tightening of $AuCl_3$ vs. TPMG4, (c) magnified view of UV-Vis absorption spectrum near the isoabsorption point and (d) the change in absorbance at 287 nm.

Figure S3. (a) STEM image, (b) STEM/EDS mapping image and (c,d) EDS spectrum (yellow: whole image, gray: blank area) of $Au_{25}PET_{18}$

Figure S4. XPS spectrum of $Au_{25}PET_{18}$ and Au wire. X-ray source is monochrome Al Kα (1486.7 eV). Neutralizer's emission current is 1μA.

Figure S5. MALDI-TOF-MS spectra after 2 hours of the $Au_{25}PET_{18}$ synthesis reaction with TPM G4.

Figure S6. Synthetic procedure of $Au_{25}PET_{18}$ cluster not using TPM G4. Note: main products is Au nanoparticle. The concentration of $AuCl_3$ was the same as when synthesized using TPM G4. In the presence of dendrimers, the equivalent of 25 equivalents of $AuCl_3$ is 45 μM.

Figure S7. MALDI-TOF-MS spectra after 2 hours of the $Au_{25}PET_{18}$ synthesis reaction without TPM G4.

Figure S8. Comparison during synthesis of $Au_{25}PET_{18}$ with and without TPM G4. A significant color change is observed immediately after reduction.

Figure S9. Image of the effect of dendrimer during $Au_{25}PET_{18}$ synthesis and its main and byproducts.

Figure S10. The image of the stable structures of $PdAu_{24}PET_{18}$ (note: draw a figure with reference to the previous report [76]).

Figure S11. (a) STEM image, (b) STEM/EDS mapping image and (c) EDS spectrum (purple: whole image, gray: blank area) of $PdAu_{24}PET_{18}$.

Figure S12. (a) Synthetic scheme of thiol-protected Au clusters blended with different elements (Pt), (b) the image of the possible structures of $PtAu_{24}PET_{18}$ (note: draw a figure with reference to the previous report [20],), (c) MS spectrum of $PtAu_{24}SR_{18}$.

Figure S13. (a) STEM image, (b) STEM/EDS mapping image and (c,d) EDS spectrum (light blue: whole image, gray: blank area) of $PtAu_{24}PET_{18}$.

Figure S14. A comparison of the simulated values (light blue: $PtAu_{24}PET_{18}$ and yellow: $Au_{25}PET_{18}$) and the measured value of $PtAu_{24}PET_{18}$ (red).

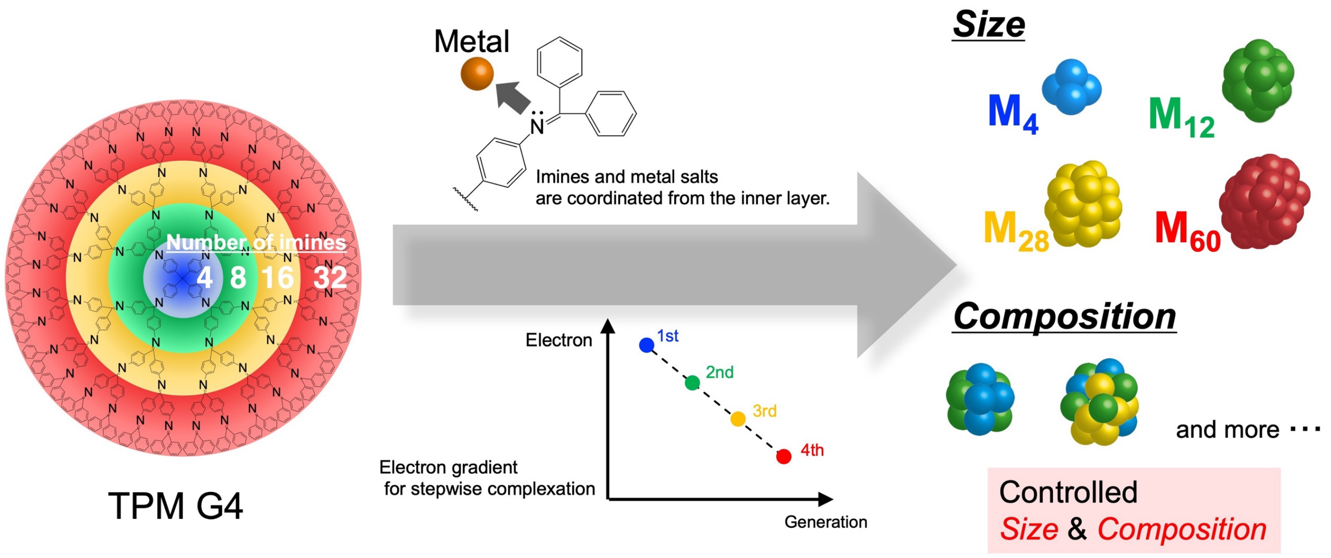

Figure S1. Schematic diagram of the structure and coordination of the dendrimer (TPM G4) used in this study.

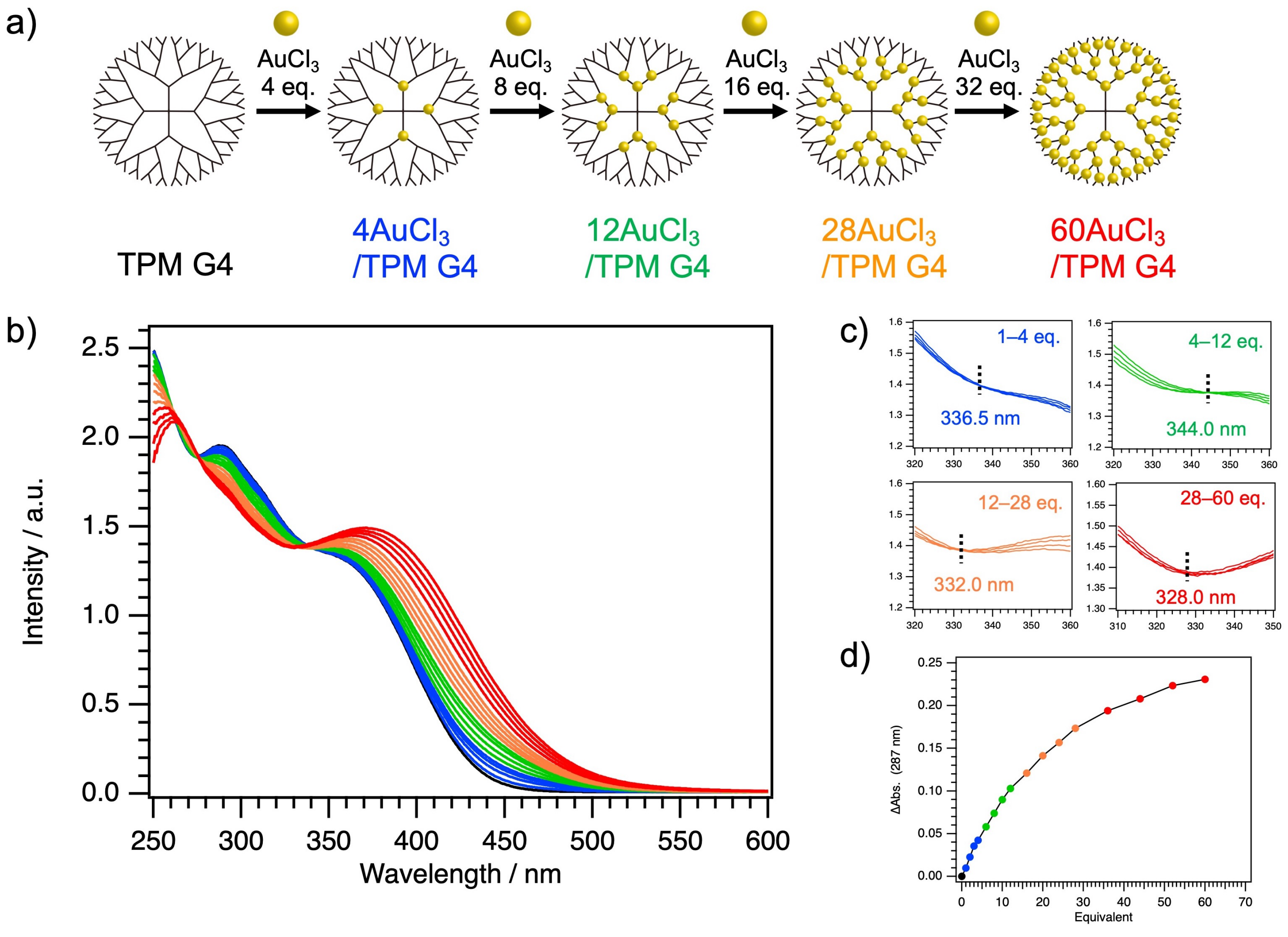

Figure S2. (a) Coordination image of $AuCl_3$ to TPMG4, (b) UV-Vis absorption spectra obtained by tightening of $AuCl_3$ vs. TPMG4, (c) magnified view of UV-Vis absorption spectrum near the isoabsorption point and (d) the change in absorbance at 287 nm.

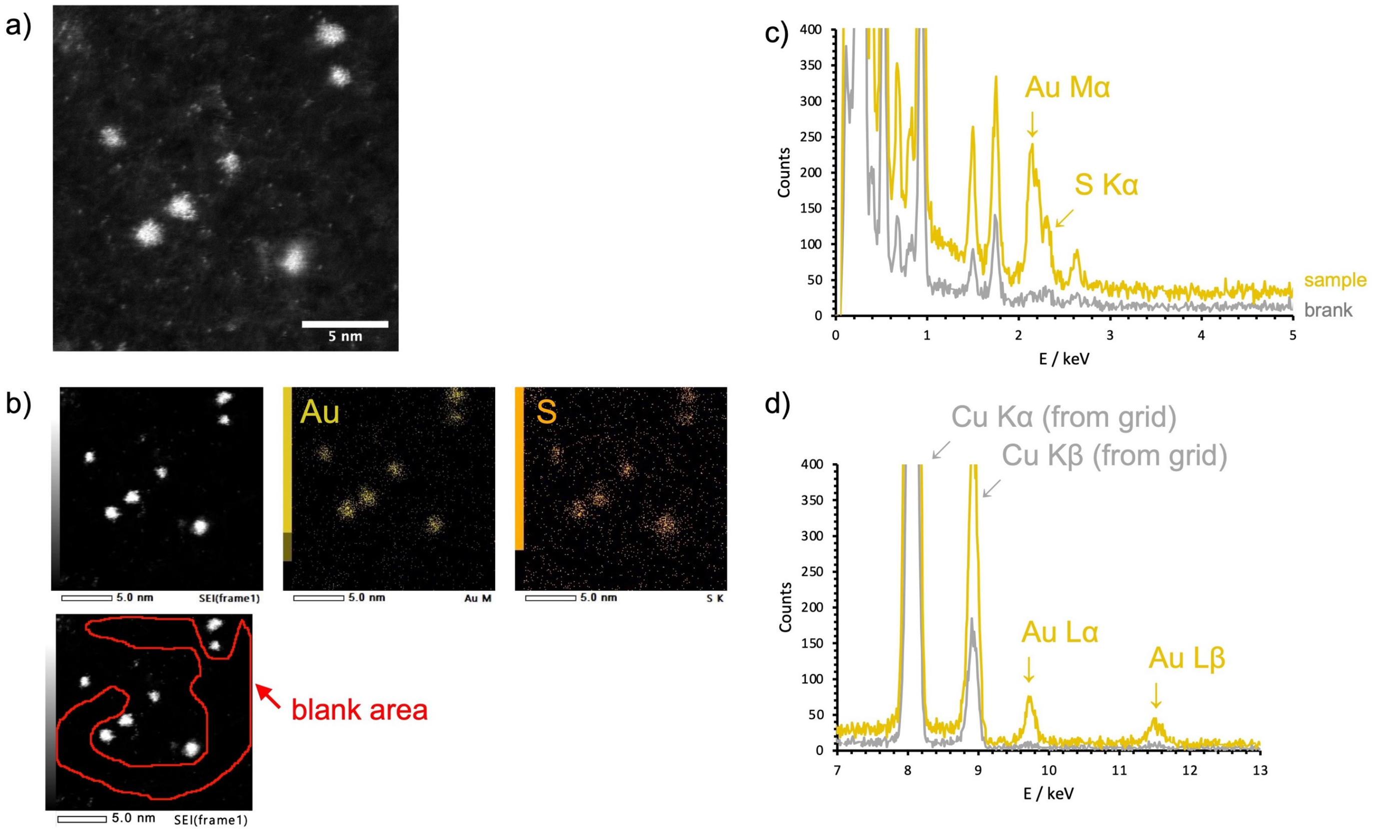

Figure S3. (a) STEM image, (b) STEM/EDS mapping image and (c,d) EDS spectrum (yellow: whole image, gray: blank area) of $Au_{25}PET_{18}$

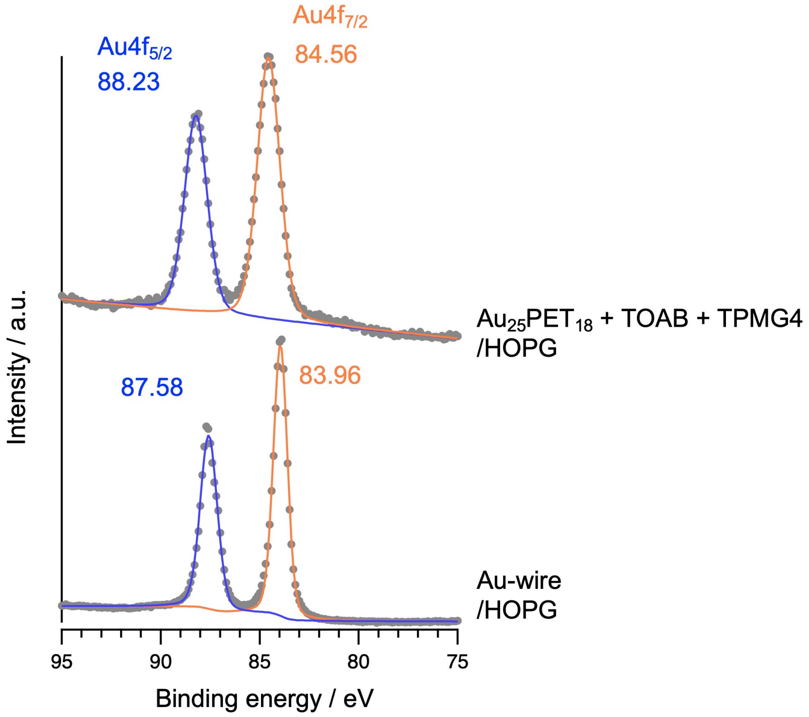

Figure S4. XPS spectrum of $Au_{25}PET_{18}$ and Au wire. X-ray source is monochrome Al Kα (1486.7 eV). Neutralizer's emission current is 1μA.

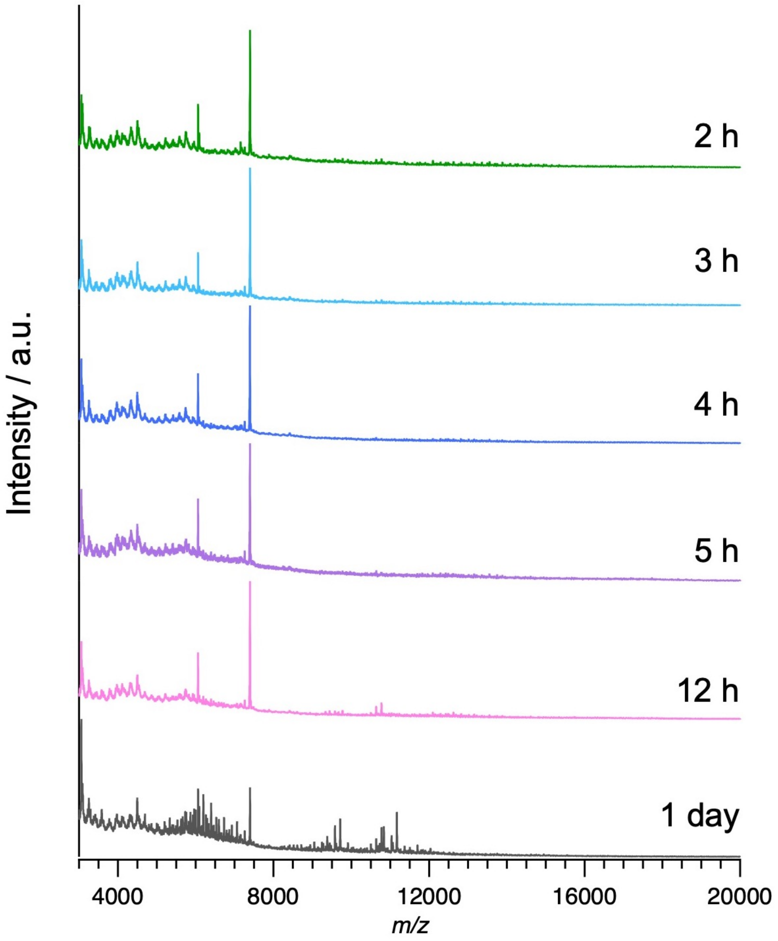

Figure S5. MALDI-TOF-MS spectra after 2 hours of the $Au_{25}PET_{18}$ synthesis reaction with TPM G4.

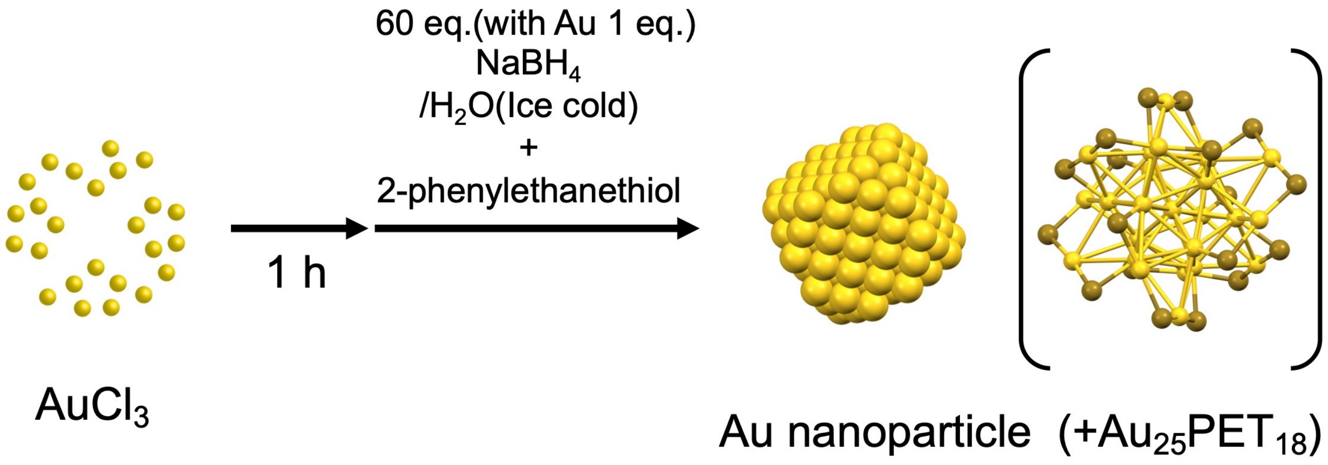

Figure S6. Synthetic procedure of $Au_{25}PET_{18}$ cluster not using TPM G4. Note: main products is Au nanoparticle. The concentration of $AuCl_3$ was the same as when synthesized using TPM G4. In the presence of dendrimers, the equivalent of 25 equivalents of $AuCl_3$ is 45 μM.

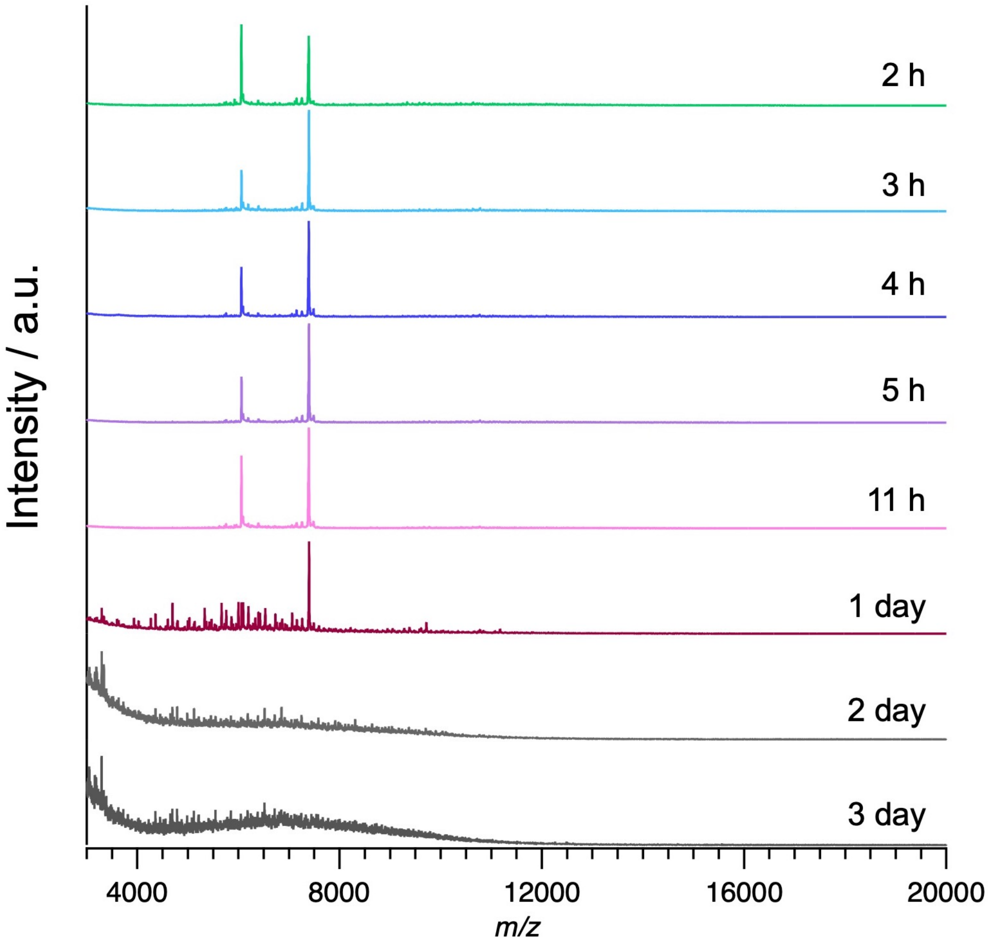

Figure S7. MALDI-TOF-MS spectra after 2 hours of the $Au_{25}PET_{18}$ synthesis reaction without TPM G4.

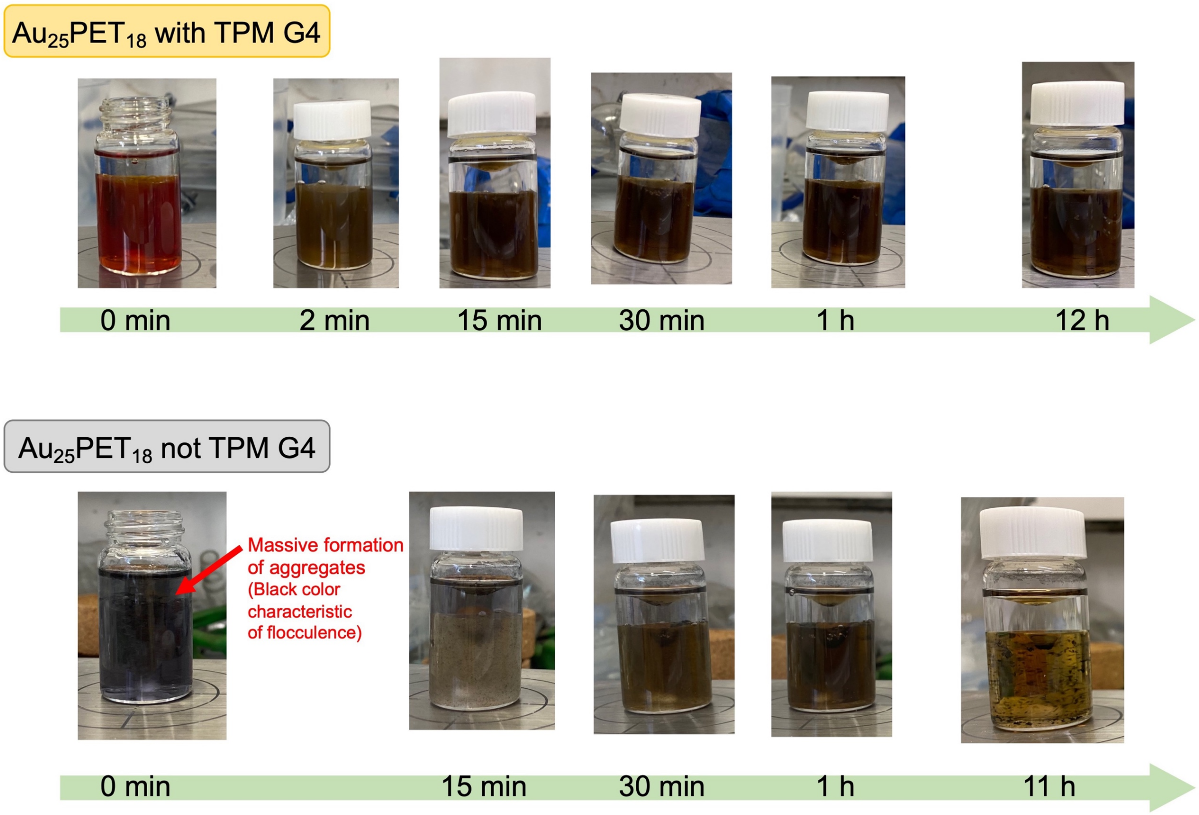

Figure S8. Comparison during synthesis of $Au_{25}PET_{18}$ with and without TPM G4. A significant color change is observed immediately after reduction.

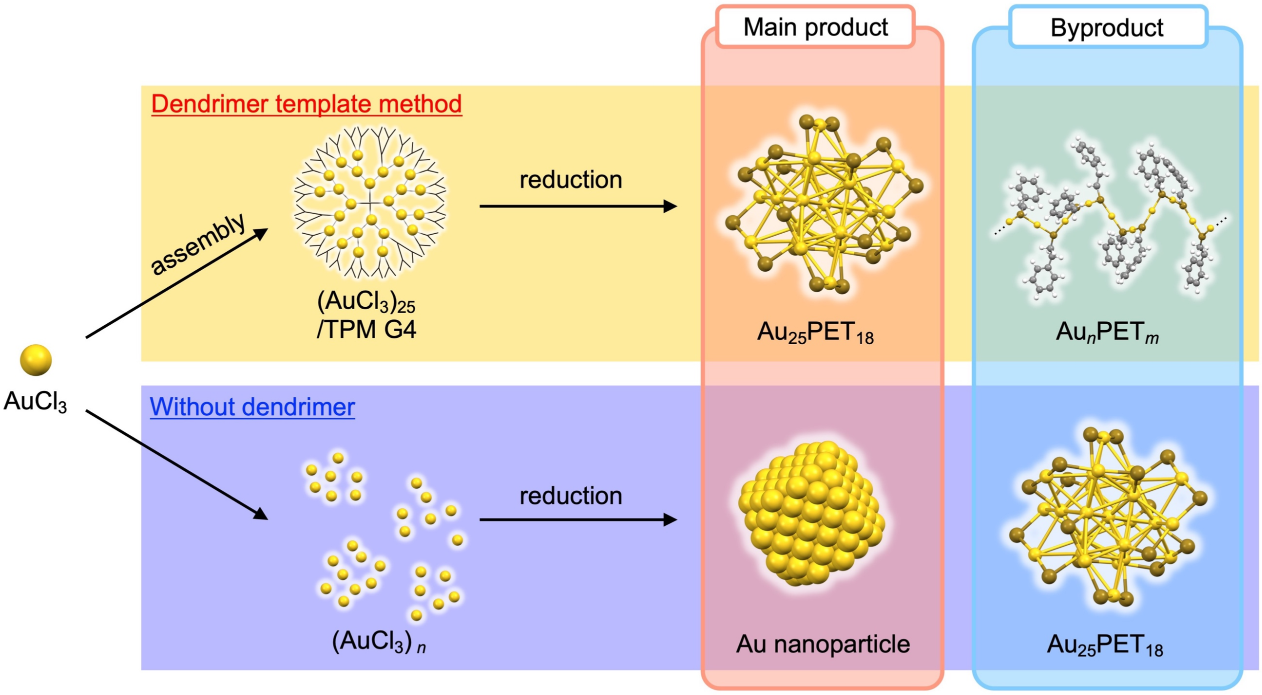

Figure S9. Image of the effect of dendrimer during $Au_{25}PET_{18}$ synthesis and its main and byproducts.

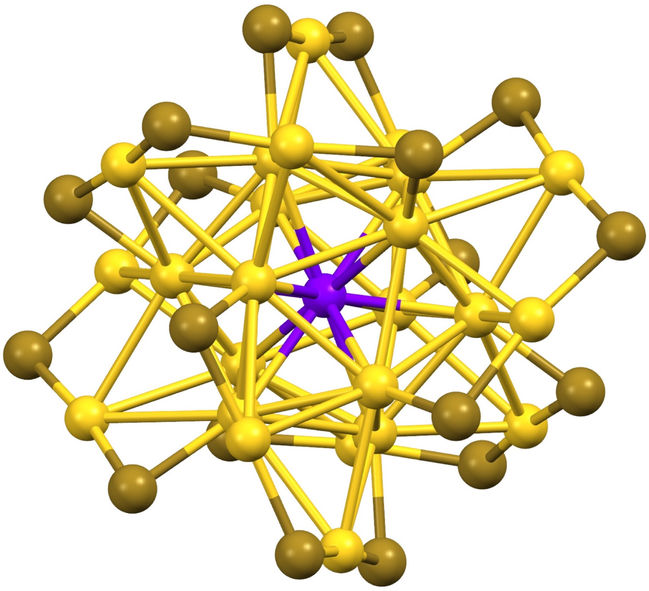

Figure S10. The image of the stable structures of $PdAu_{24}PET_{18}$ (note: draw a figure with reference to the previous report [76]).

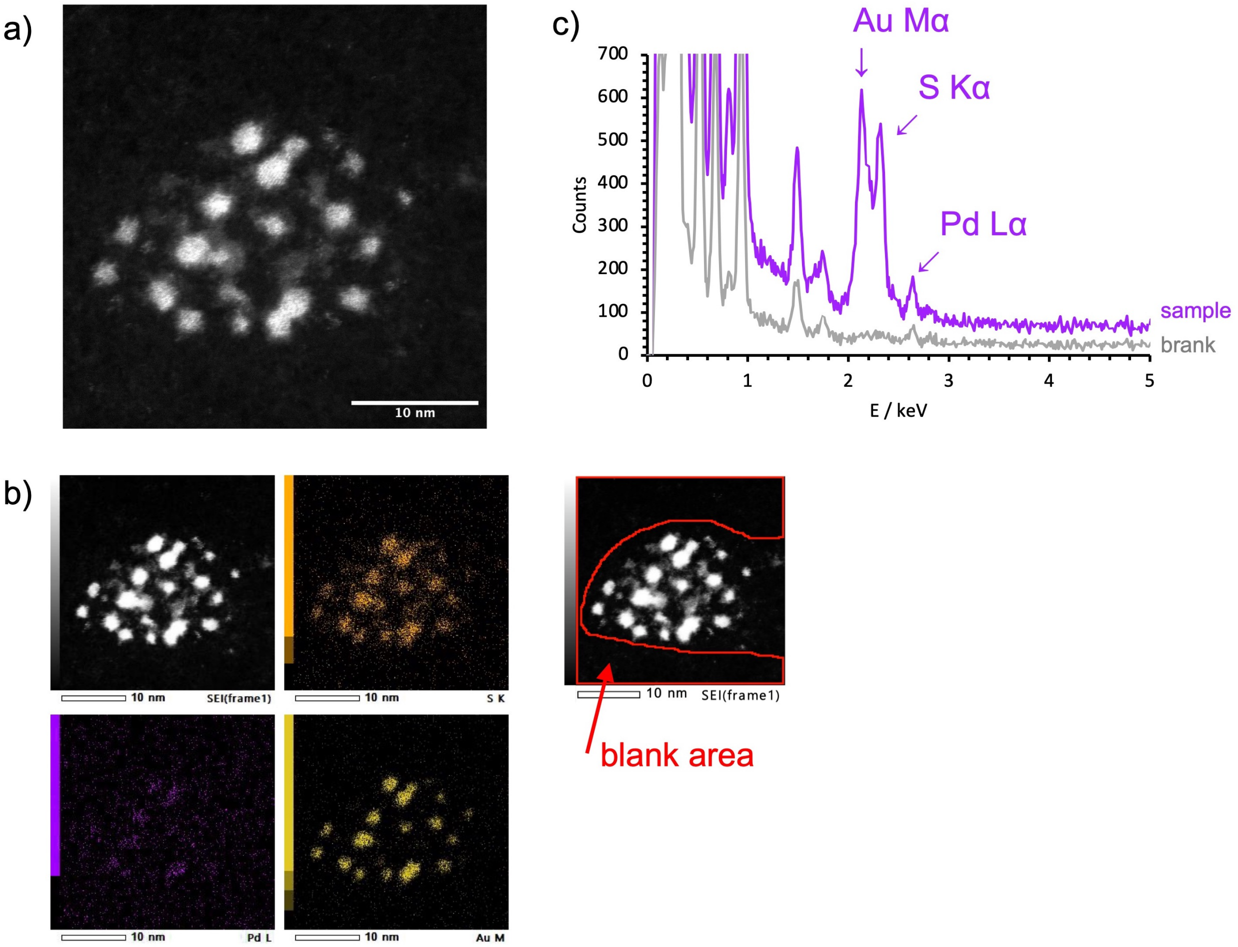

Figure S11. (a) STEM image, (b) STEM/EDS mapping image and (c) EDS spectrum (purple: whole image, gray: blank area) of $PdAu_{24}PET_{18}$.

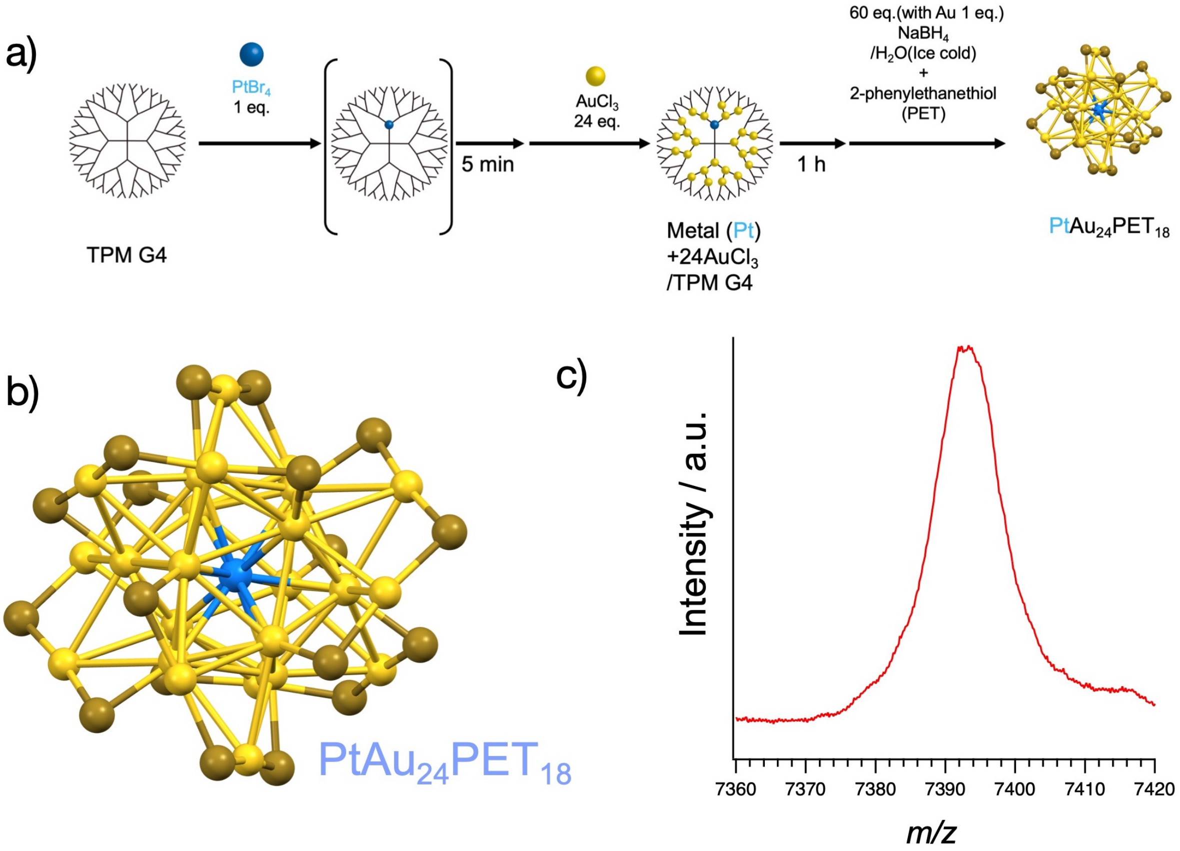

Figure S12. (a) Synthetic scheme of thiol-protected Au clusters blended with different elements (Pt), (b) the image of the possible structures of $PtAu_{24}PET_{18}$ (note: draw a figure with reference to the previous report [20],), (c) MS spectrum of $PtAu_{24}SR_{18}$.

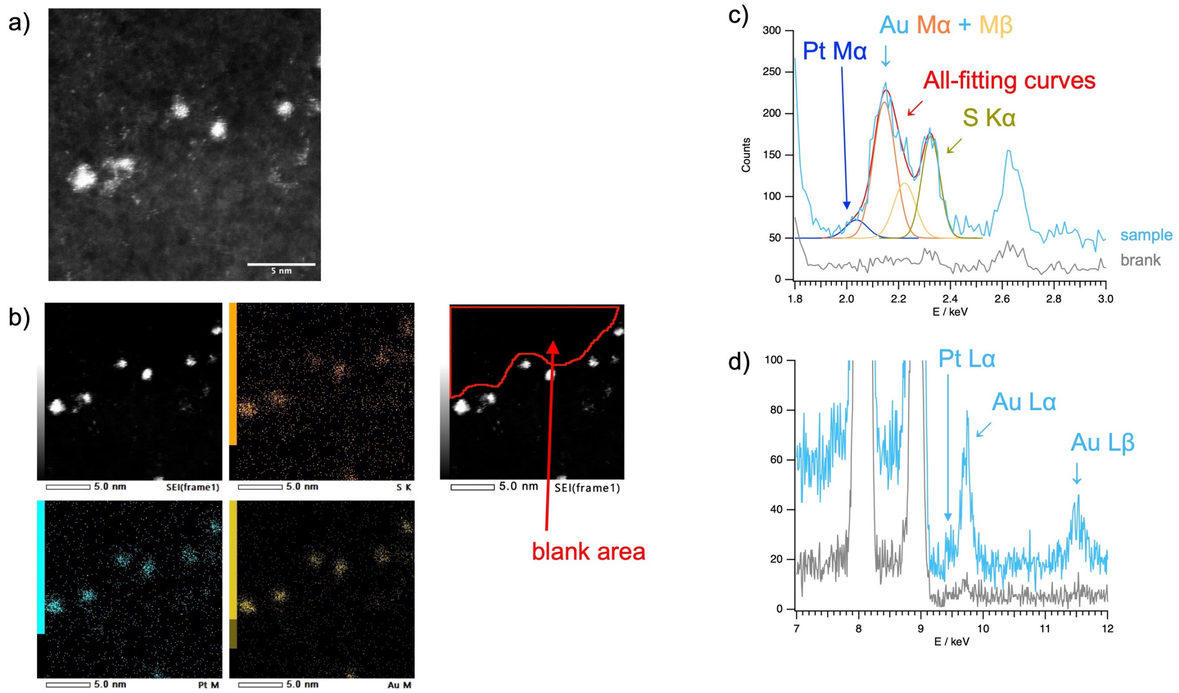

Figure S13. (a) STEM image, (b) STEM/EDS mapping image and (c,d) EDS spectrum (light blue: whole image, gray: blank area) of $PtAu_{24}PET_{18}$.

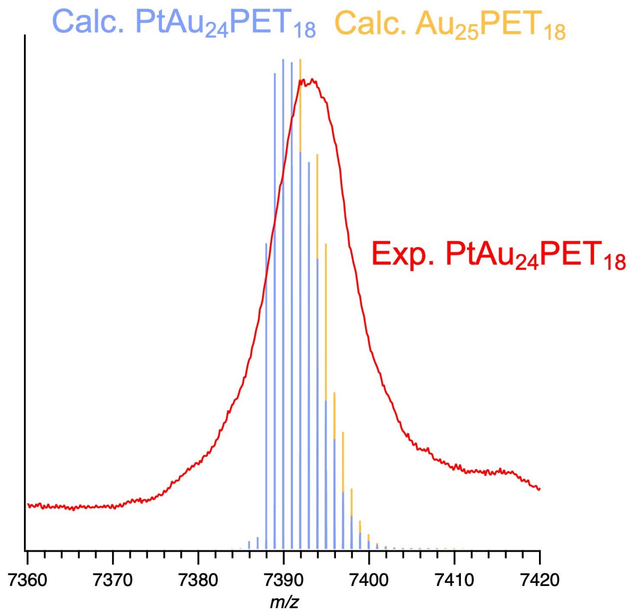

Figure S14. A comparison of the simulated values (light blue: $PtAu_{24}PET_{18}$ and yellow: $Au_{25}PET_{18}$) and the measured value of $PtAu_{24}PET_{18}$ (red).
